# Laparoscopic Resection Rectopexy with Transanal Specimen Extraction for Complete Rectal Prolapse: Retrospective Cohort Study of Functional Outcomes

**DOI:** 10.3390/jcm15020718

**Published:** 2026-01-15

**Authors:** Mustafa Ates, Sami Akbulut, Emrah Sahin, Kemal Baris Sarici, Ertugrul Karabulut, Mukadder Sanli

**Affiliations:** 1 Department of Surgery, Faculty of Medicine, Inonu University, 44280 Malatya, Türkiye; 2 Department of Anesthesiology and Reanimation, Faculty of Medicine, Inonu University, 44280 Malatya, Türkiye

**Keywords:** complete rectal prolapse, laparoscopic resection rectopexy, transanal specimen extraction, low anterior resection syndrome scores, wexner incontinence score, obstructed defecation syndrome

## Abstract

**Background:** Complete rectal prolapse (RP) is a debilitating pelvic floor disorder often accompanied by obstructed defecation syndrome (ODS), fecal incontinence, and LARS-like bowel dysfunction. Laparoscopic resection rectopexy (LRR) is an established abdominal approach; however, functional outcomes after LRR with transanal specimen extraction (LRR-TSE) are incompletely defined. **Aim**: To evaluate short- and long-term functional outcomes—ODS, Wexner incontinence score (WIS), and LARS—in patients undergoing LRR-TSE. **Methods**: This single-center cohort included 53 consecutive patients who underwent LRR-TSE between January 2013 and December 2019. Variables were prospectively recorded and analyzed retrospectively. ODS, WIS, and LARS scores were assessed preoperatively and at 3, 6, and 12 months. Longitudinal changes were analyzed using repeated-measures ANOVA with Greenhouse–Geisser correction, polynomial contrasts when appropriate, and Bonferroni-adjusted pairwise comparisons. **Results**: ODS improved significantly over time (*p* < 0.001), decreasing from 12.8 ± 3.2 preoperatively to 2.4 ± 2.1, 4.2 ± 2.2, and 5.2 ± 2.9 at 3, 6, and 12 months, respectively. LARS scores declined from 18.0 ± 12.7 at 3 months to 8.8 ± 6.8 at 6 months and 3.5 ± 4.2 at 12 months (*p* < 0.001). WIS showed a transient increase at 3 months (8.1 ± 5.2), followed by improvement at 6 and 12 months (3.2 ± 3.7 and 2.4 ± 3.0; *p* < 0.001). Sex and body mass index did not affect functional trajectories (*p* > 0.05), whereas patients aged ≥50 years had higher postoperative LARS and WIS scores (*p* < 0.05). Complications occurred in 5 patients (9.43%), including one anastomotic leak with a mortality rate of 1.85%. Full-thickness recurrence occurred in 2 patients (3.77%), and 3 developed mucosal prolapse managed with Delorme’s procedure. **Conclusions**: LRR-TSE is a safe and feasible minimally invasive technique that improves constipation, continence, and LARS-related bowel dysfunction. Early postoperative impairment may overestimate long-term functional severity, highlighting the need for follow-up beyond 12 months.

## 1. Introduction

Rectal prolapse (RP) is broadly classified as external or internal. External (full-thickness) RP, also termed complete RP, is defined by a circumferential, full-thickness protrusion of the rectal wall through the anal canal. In contrast, internal RP (rectal intussusception) involves intraluminal telescoping of the rectal wall without external protrusion and represents a distinct entity with different pathophysiological and therapeutic implications [1,2,3,4]. Although its etiology is multifactorial, RP is strongly associated with posterior pelvic compartment disorders, including rectocele, enterocele, and multicompartment pelvic organ prolapse, supporting the concept of a global pelvic floor dysfunction rather than an isolated anatomic defect [5,6,7,8,9,10,11]. Clinically, RP presents with rectal discomfort, bleeding, fecal incontinence—reported in up to 88% of patients—and obstructed defecation syndrome (ODS), all of which substantially impair quality of life [12,13,14,15,16]. Accordingly, postoperative functional recovery is most appropriately assessed using validated instruments such as the ODS score, the Wexner Incontinence Score (WIS), and the Low Anterior Resection Syndrome (LARS) score to quantify LARS-like bowel dysfunction [17].

Epidemiological data demonstrate marked geographic variation in RP incidence, ranging from 18.5 per 100,000 in England—approximately sevenfold higher than in Finland—to 2.5–4.2 per 100,000 in broader population-based analyses [10,11,18,19]. Despite this variability, the disease shows a consistent demographic pattern characterized by a bimodal age distribution and a pronounced female predominance, with elderly women accounting for 80–90% of cases [10,11,18,19,20,21]. This demographic profile continues to inform contemporary diagnostic and therapeutic strategies.

Surgery remains the definitive treatment for RP [10]. Available techniques include perineal procedures and transabdominal approaches performed using open, laparoscopic, or robotic platforms [10]. Among abdominal operations, laparoscopic and robotic resection rectopexy have gained increasing acceptance because of favorable functional outcomes and lower recurrence rates compared with open surgery [1,22,23]. Nevertheless, most published series report conventional laparoscopic resection rectopexy (LRR) with abdominal specimen extraction [24,25,26,27,28,29,30], whereas evidence regarding natural orifice specimen extraction (NOSE) techniques remains limited [31,32,33].

NOSE has been selectively incorporated into colorectal surgery to reduce abdominal wall morbidity. Meta-analyses and large series have demonstrated reduced postoperative pain, fewer wound-related complications, and improved cosmetic outcomes when appropriate contamination-control measures are applied [34,35,36,37,38,39,40]. Despite these advantages, adoption of NOSE is heterogeneous and largely dependent on institutional expertise and patient selection, and its application in RP surgery has been confined to small case series. Consequently, the functional outcomes of laparoscopic resection rectopexy with transanal specimen extraction (LRR-TSE) in RP have not been systematically evaluated.

Given the limited evidence on LRR-TSE and the growing emphasis on enhanced recovery, organ-preserving dissection, and reduced port-site morbidity, further focused investigation is warranted. Therefore, the present study aims to assess short- and long-term functional outcomes in patients with complete RP undergoing LRR-TSE, with particular attention to constipation, continence, and postoperative bowel dysfunction.

## 2. Material and Methods

### 2.1. Study Design and Patient Selection

This study was conducted as an observational cohort including patients who underwent surgery at Inonu University Faculty of Medicine (Malatya, Türkiye) between January 2013 and December 2019 for complete RP. During routine clinical care, demographic (age, sex, body mass index, ASA score), clinical, endoscopic, radiological, perioperative, and postoperative data were prospectively recorded in institutional databases and subsequently retrieved and analyzed retrospectively. All consecutive patients presenting with complete RP during the study period were screened; 73 patients were evaluated, and 53 met the predefined inclusion criteria and underwent laparoscopic resection rectopexy with transanal specimen extraction (LRR-TSE). All LRR-TSE procedures were performed by the same senior surgeon to ensure technical consistency, who had a minimum of five years of experience in laparoscopic abdominal, colorectal, and pelvic floor surgery at study initiation.

Complete RP was diagnosed by standardized clinical examination and defined as a circumferential, full-thickness protrusion of the rectal wall through the anal canal, confirmed by direct visualization during straining or the Valsalva maneuver in the seated position [13,41]. All patients underwent preoperative colonoscopy to exclude concomitant colonic pathology that could influence surgical planning or functional outcomes [13,41]. In addition, all female patients were routinely evaluated by a gynecologist to assess multicompartment pelvic organ prolapse, in line with recommendations for multidisciplinary pelvic floor assessment [13].

### 2.2. Inclusion and Exclusion Criteria

Patients were eligible for inclusion if they met the following criteria: (i) full-thickness RP measuring ≥5 cm, (ii) American Society of Anesthesiologists (ASA) class I–III, and (iii) suitability for minimally invasive abdominal surgery. Exclusion criteria comprised (i) multicompartment pelvic organ prolapse requiring alternative reconstructive strategies; (ii) solitary rectal ulcer syndrome, rectocele, or enterocele; (iii) RP < 5 cm; and (iv) ASA class ≥ IV. Patients who did not meet the inclusion criteria were managed with alternative surgical approaches, such as ventral mesh rectopexy or the Delorme procedure.

### 2.3. Scoring Systems

#### 2.3.1. Low Anterior Resection Syndrome (LARS) Score

The LARS score was developed to evaluate postoperative bowel dysfunction following low anterior resection and includes five domains: incontinent flatus, liquid stool incontinence, bowel movement frequency, stool clustering, and urgency. Scores are categorized as no LARS (0–20), minor LARS (21–29), or major LARS (30–42) [42,43]. Although patients did not undergo oncological low anterior resection, the LARS score was applied as a standardized measure of LARS-like bowel dysfunction after LRR-TSE.

#### 2.3.2. Wexner Incontinence Score (WIS)

The Wexner Incontinence Score (WIS), also known as the Cleveland Clinic Fecal Incontinence Severity Scoring System (CCIS), assesses fecal incontinence across five domains: incontinence to solid stool, liquid stool, and gas; pad use; and lifestyle alteration. Each domain is scored from 0 to 4, yielding a total score of 0–20, with higher scores indicating more severe incontinence; scores are commonly classified as mild (1–3), moderate (4–8), or severe (9–20) [41,44,45,46].

#### 2.3.3. Obstructed Defecation Syndrome (ODS) Score

The ODS score is a validated instrument used to quantify symptoms of obstructed defecation, including excessive straining, incomplete evacuation, laxative or enema dependence, need for digital maneuvers, and defecation-related abdominal discomfort. Each item is rated on a five-point Likert scale (0–4), yielding a cumulative score of 0–20, with higher values indicating greater symptom severity [47].

### 2.4. Study Groups

Patients were stratified by sex (female vs. male), body mass index (BMI < 25 vs. ≥25 kg/m^2^), and age (<50 vs. ≥50 years). These predefined subgroups were compared for baseline characteristics, perioperative outcomes, and postoperative functional trajectories, allowing assessment of overall and subgroup-specific changes in ODS, WIS, and LARS scores.

### 2.5. Surgical Technique

All patients received standardized perioperative preparation, including mechanical bowel cleansing, antibiotic prophylaxis, and antithrombotic prophylaxis [48,49]. Procedures were performed under general anesthesia in the Lloyd–Davis position using four laparoscopic ports; in female patients, the uterus was temporarily suspended to the anterior abdominal wall to improve pelvic exposure.

**Figure 1:**
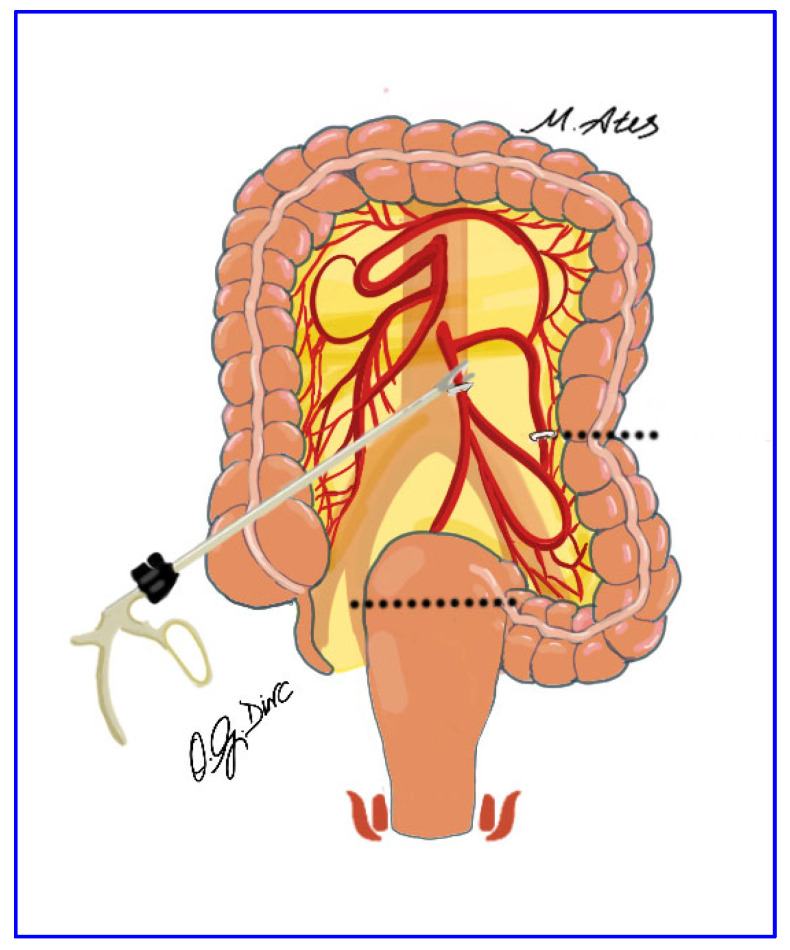
Ligation of the inferior mesenteric artery at its origin using a vessel-sealing device, with adjunct hemoclips applied for secure hemostasis.

The procedure began with incision of the right leaf of the sigmoid mesocolon and entry into the avascular plane between the visceral and parietal pelvic fascia approximately 2 cm anterior to the sacral promontory. The left ureter and gonadal vessels were identified and preserved. High ligation of the inferior mesenteric artery was performed approximately 3 cm from its origin using vascular sealing devices or hemoclips, with preservation of the superior hypogastric plexus (Figure 1). Dissection continued with division of the inferior mesenteric vein and mobilization of the mesorectum within the avascular presacral plane, preserving the superior hypogastric nerves, their pelvic branches, and the lateral rectal ligaments. Anterior dissection was carried out to open the Douglas pouch to a depth of approximately 1 cm.

**Figure 2:**
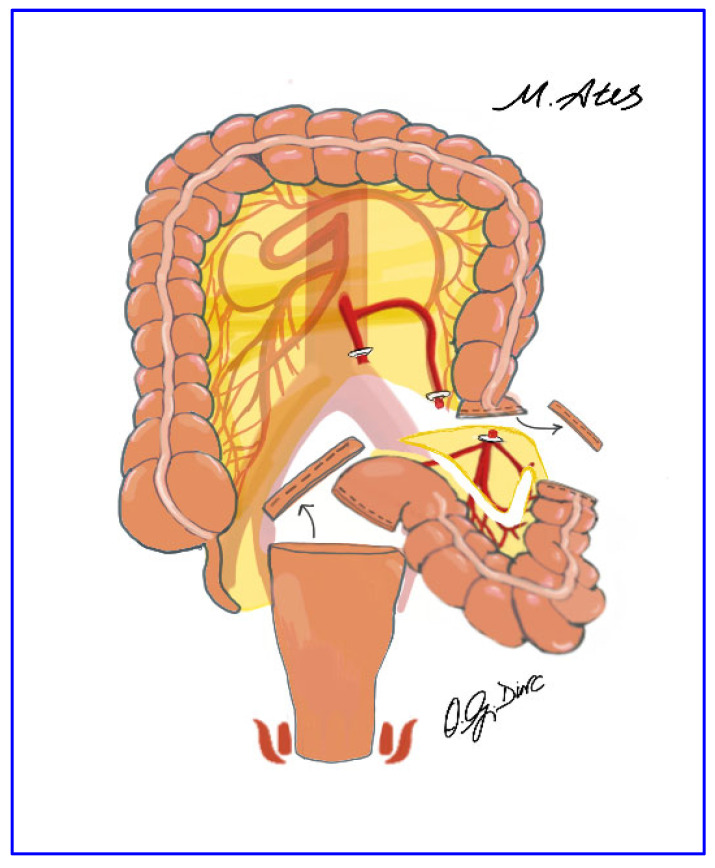
Division of the distal descending colon with a laparoscopic endo-linear stapler applied perpendicular to the bowel axis.

**Figure 3:**
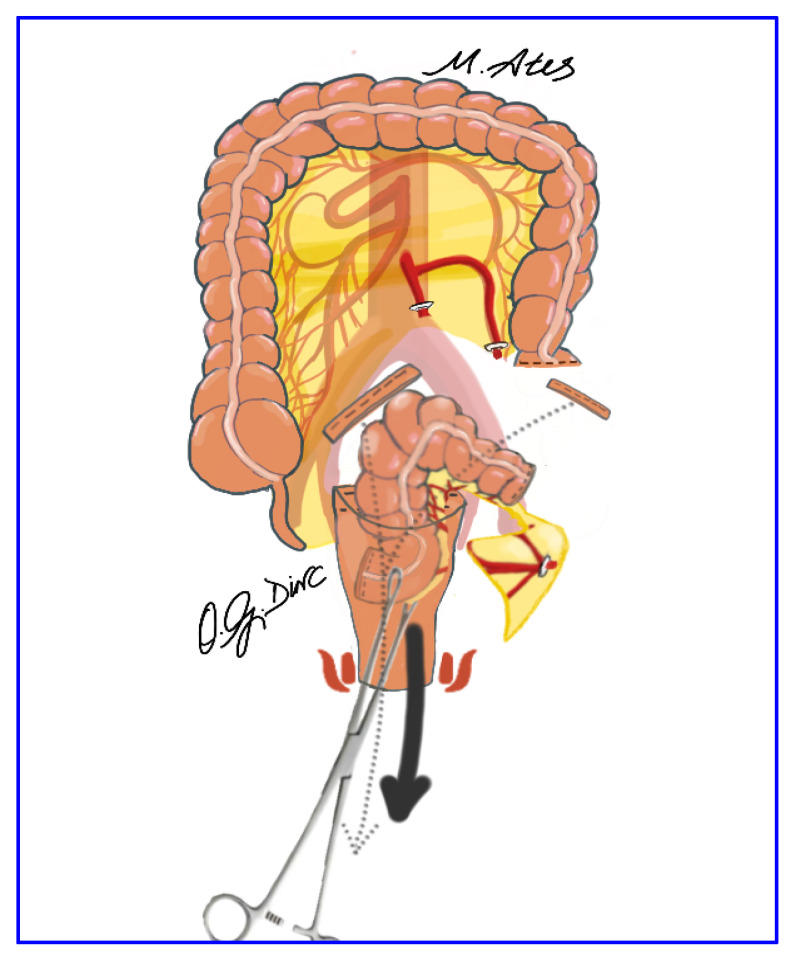
Transanal extraction of the resected mesosigmoid specimen using long sponge forceps.

After pelvic dissection, the redundant sigmoid colon was elevated to determine the resection level at the sacral promontory. Resection and division of the distal descending colon were performed using a laparoscopic endo-linear stapler, leaving the specimen free within the peritoneal cavity (Figure 2). The rectum was irrigated with diluted povidone–iodine solution before and after transection. The distal rectal stump was opened transversely, allowing transanal introduction of long sponge forceps for specimen extraction; mesenteric division with a vessel-sealing device was performed when necessary (Figure 3). Additional mobilization, including parietal or omental division or splenic flexure release, was undertaken as required.

**Figure 4:**
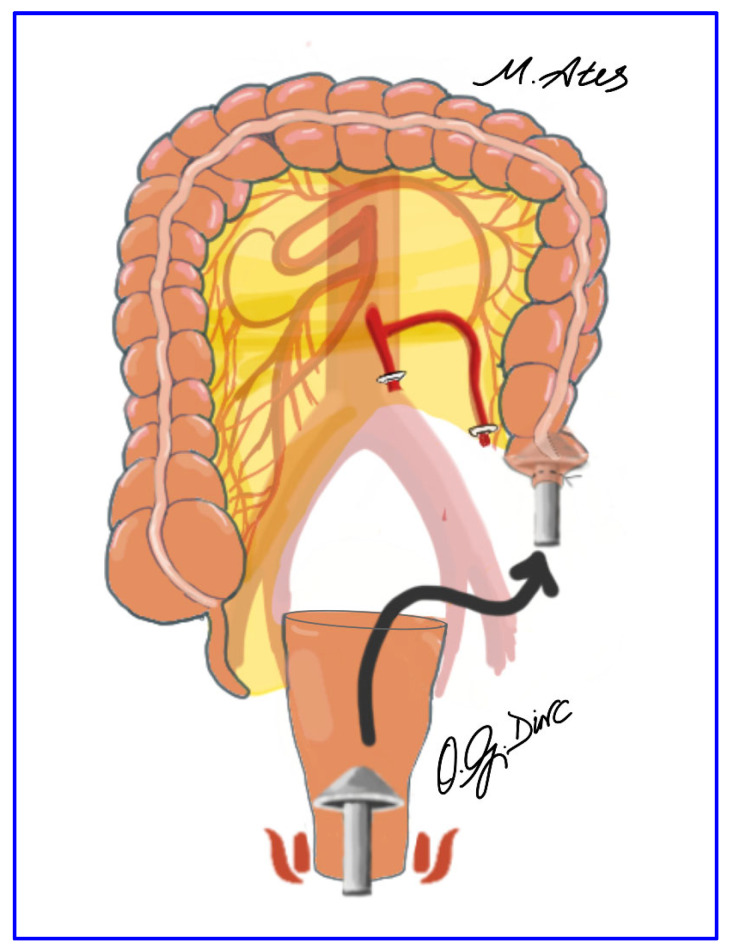
Transanal introduction of the circular stapler anvil into the pelvic cavity under direct laparoscopic visualization.

**Figure 5:**
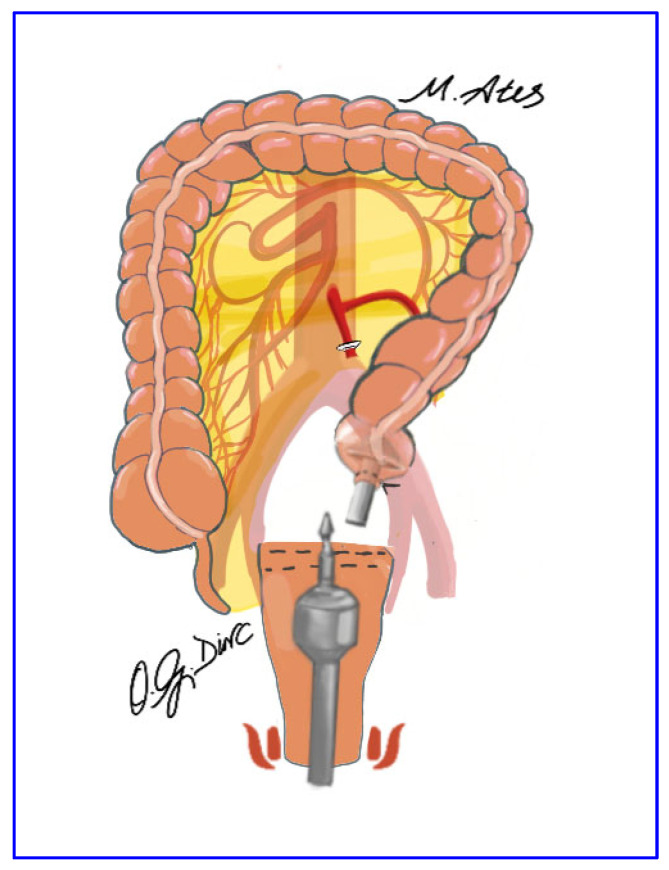
Intracorporeal hand-sewn purse-string suture placement on the distal descending colon, followed by closure of the proximal rectal stump with a laparoscopic linear stapler.

**Figure 6:**
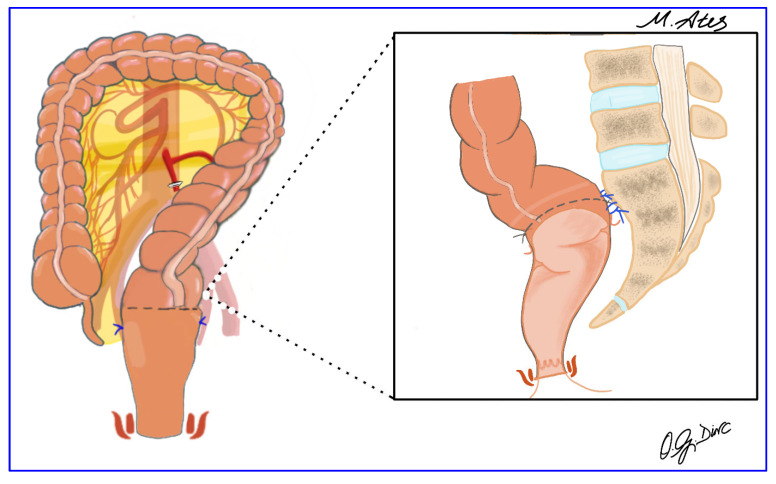
Rectopexy to the sacral promontory using 2-0 non-absorbable sutures after completion of colorectal anastomosis.

The anvil of a circular stapler was introduced transanally into the pelvis (Figure 4). After transverse opening of the stapler line of the distal descending colon, an intracorporeal hand-sewn purse-string suture was placed, and the short stapled cuff was removed transanally. The proximal rectal stump was closed using a laparoscopic linear stapler (Figure 5), and the pelvis was irrigated with 0.9% saline. Rectopexy was performed with 2-0 nonabsorbable sutures anchoring the rectum to the sacral promontory, followed by intracorporeal colorectal anastomosis using the double-stapling technique. A pelvic drain was routinely placed to complete the procedure (Figure 6).

### 2.6. Follow Up

Functional assessments were performed at 3, 6, and 12 months postoperatively using the ODS, WIS, and LARS scoring systems. Evaluations were primarily conducted during in-person outpatient visits; when this was not feasible, standardized remote assessments were used. Recurrence was defined as full-thickness RP confirmed by physical examination or, when in-person evaluation was not possible, by structured symptom-based reporting focused on protrusion during straining. After the 12-month evaluation, patients underwent annual follow-up, either in clinic or via structured remote assessment, for long-term recurrence surveillance.

### 2.7. Study Protocol, Ethical Approval, and Funding

This study was conducted in accordance with the Declaration of Helsinki and applicable institutional and national guidelines. Ethical approval was obtained from the Inonu University Institutional Review Board for non-interventional studies (Approval Number: 3594; 21 June 2022). The study protocol, data collection, analysis, and reporting adhered to the STROBE guidelines to ensure methodological rigor and transparency. No external funding was received, and the study was supported by institutional resources.

### 2.8. Statistical Analysis

Statistical analyses were performed using SPSS version 25.0 (IBM SPSS Statistics, Armonk, NY, USA). Normality of continuous variables was assessed using the Kolmogorov–Smirnov test. Continuous variables are presented as mean ± standard deviation (SD) with 95% confidence intervals (CIs) and categorical variables as frequencies and percentages. Comparisons between two independent groups (sex, BMI category, and age category) were performed using the independent-samples Student’s *t*-test for continuous variables, with variance equality assessed by Levene’s test, and the chi-square or Fisher’s exact test for categorical variables, as appropriate.

Longitudinal changes in functional outcomes (ODS, LARS, and WIS) across four time points (preoperative, POD90, POD180, and POD365) were analyzed using repeated-measures analysis of variance (RM-ANOVA) with Greenhouse–Geisser correction. Analyses were conducted for the entire cohort and predefined subgroups (sex, BMI, and age), evaluating within-subject time effects, between-group differences, and time × group interactions. When significant time effects were identified, polynomial contrasts (linear, quadratic, and cubic) were applied to characterize temporal trajectories, and Bonferroni-adjusted pairwise comparisons based on estimated marginal means were used to identify specific differences. A two-sided *p*-value < 0.05 was considered statistically significant. Effect sizes were expressed as partial eta squared (η^2^p). Sphericity was assessed using Mauchly’s test, with Greenhouse–Geisser epsilon (ε) corrections applied when assumptions were violated. Post hoc statistical power for temporal effects was estimated based on observed η^2^p values, sample size, number of repeated measurements, and F-GG–adjusted degrees of freedom.

We used artificial intelligence tools solely for language editing and grammatical improvement of the manuscript. Artificial intelligence was not used for study design, data analysis, methodology development, or interpretation of the results.

## 3. Results

### 3.1. Baseline Demographic and Clinical Characteristics of the Entire Study Cohort

A total of 53 patients who underwent LRR-TSE were included in the analysis. The mean age was 45 ± 14 years; 32 patients (60.4%) were female and 21 (39.6%) were male. The mean BMI was 27.1 ± 3.5 kg/m^2^. One patient had previously undergone laparoscopic mesh rectopexy for RP. Preoperatively, the mean ODS score was 12.8 ± 3.2, with 44 patients (83.0%) exhibiting moderate to severe symptoms. The mean preoperative WIS score was 4.0 ± 3.9; 25 patients (47.2%) had mild, 21 (39.6%) moderate, and 7 (13.2%) severe incontinence. According to ASA classification, 36 patients (67.9%) were ASA I and 17 (32.1%) ASA II. The mean specimen length was 25 ± 7 cm, the mean hospital stay was 5 ± 4 days, and the mean follow-up duration was 79.6 ± 19.5 months.

### 3.2. Longitudinal Changes in ODS, LARS, and WIS Scores Across the Entire Study Cohort

ODS scores changed significantly over time (F-GG–corrected RM-ANOVA: F(1.82, 94.51) = 322.81, *p* < 0.001; partial η^2^ = 0.861). All time points differed after Bonferroni correction (*p* < 0.001 for all). Preoperative scores were higher than POD90, POD180, and POD365 (mean differences: +10.43, +8.57, and +7.57). POD90 was the lowest postoperative value and differed from POD180 and POD365 (mean differences: −1.87 and −2.87). POD180 and POD365 also differed, with higher scores at POD365 (mean difference: −1.00) (Figure S1). LARS scores also changed significantly over time (F-GG-corrected RM-ANOVA: F(1.27, 65.83) = 80.03, *p* < 0.001; partial η^2^ = 0.606). All measurement points differed after Bonferroni adjustment (*p* < 0.001 for all). Preoperative scores were higher than POD180 and POD365 (mean differences: +9.19 and +14.45), and POD180 exceeded POD365 (mean difference: +5.26), indicating a progressive postoperative decline (Figure S2). WIS scores changed significantly over time (F-GG-corrected RM-ANOVA: F(1.92, 99.94) = 70.11, *p* < 0.001; partial η^2^ = 0.574). POD90 values were higher than all other time points (all Bonferroni-adjusted *p* < 0.001). Preoperative WIS did not differ from POD180 (*p* = 0.128) but was higher than POD365 (*p* < 0.001). POD180 and POD365 also differed (*p* = 0.003), with the lowest scores at POD365 (Figure S3).

### 3.3. Sex-Based Comparative Analysis of Clinical and Functional Outcomes

Female patients were older than male patients (*p* = 0.024), whereas ASA distribution (*p* = 0.374), BMI (*p* = 0.544), and length of hospital stay (*p* = 0.529) were comparable between sexes. Preoperative ODS and WIS scores did not differ significantly by sex (*p* = 0.125 and *p* = 0.874, respectively). Postoperative ODS scores were comparable between females and males at POD90 (*p* = 0.148), POD180 (*p* = 0.732), and POD365 (*p* = 0.981), as were LARS scores at POD90 (*p* = 0.694), POD180 (*p* = 0.836), and POD365 (*p* = 0.341). Postoperative WIS scores differed between sexes only at POD90 (*p* = 0.037) but were similar at POD180 and POD365 (*p* = 0.407 and *p* = 0.573, respectively). Follow-up duration was comparable between groups (*p* = 0.312) (Table 1).

### 3.4. Sex-Based Longitudinal Changes in ODS, LARS and WIN Scores

ODS scores were high preoperatively in both sexes, decreased markedly at POD90, increased partially at POD180, and stabilized at similar levels by POD365. Time had a strong effect (F-GG: F(1.80, 91.92) = 334.7, *p* < 0.001; partial η^2^ = 0.868). The main effect of sex was not significant (F(1, 51) = 0.031, *p* = 0.860; partial η^2^ = 0.001), and the time × sex interaction did not reach significance (F-GG: F(1.80, 91.92) = 3.11, *p* = 0.054; partial η^2^ = 0.058), indicating largely parallel trajectories between sexes (Figure S4). LARS scores improved over time in both sexes, with a significant main effect of time (F-GG: F(1.26, 64.39) = 74.80, *p* < 0.001; partial η^2^ = 0.595). The time × sex interaction was not significant (F-GG: F(1.26, 64.39) = 0.10, *p* = 0.813; partial η^2^ = 0.002), and no between-subjects main effect of sex was observed (F(1, 51) = 0.238, *p* = 0.628; partial η^2^ = 0.005), demonstrating similar recovery patterns in men and women (Figure S5). WIS scores changed significantly over time (F-GG: F(2.01, 102.49) = 65.05, *p* < 0.001; partial η^2^ = 0.561), with an early increase at POD90 followed by progressive improvement toward POD365. The time × sex interaction was significant (F-GG: F(2.01, 102.49) = 4.67, *p* = 0.011; partial η^2^ = 0.084), reflecting a more pronounced POD90 peak in females; however, the overall main effect of sex was not significant (F(1, 51) = 1.31, *p* = 0.258; partial η^2^ = 0.025). Thus, WIS showed transient postoperative worsening with similar long-term outcomes across sexes (Figure S6).

### 3.5. BMI-Based Comparative Analysis of Clinical and Functional Outcomes

Patients with BMI < 25 kg/m^2^ and BMI ≥ 25 kg/m^2^ demonstrated comparable baseline characteristics with respect to age (*p* = 0.739), sex distribution (*p* = 0.417), and ASA physical status (*p* = 1.000). Length of hospital stay was also similar between BMI categories (*p* = 0.148). Preoperative functional status did not differ significantly according to BMI, as reflected by comparable ODS (*p* = 0.212) and WIS (*p* = 0.429) scores. Postoperatively, functional outcomes remained similar between groups throughout follow-up. ODS scores at POD 90 (*p* = 0.301), POD 180 (*p* = 0.306), and POD 365 (*p* = 0.850) showed no significant intergroup differences. Likewise, LARS scores at POD 90 (*p* = 0.415), POD 180 (*p* = 0.879), and POD 365 (*p* = 0.243) were comparable between BMI groups. Postoperative WIS scores were comparable at POD 90 (*p* = 0.739), POD 180 (*p* = 0.210), and POD 365 (*p* = 0.173). Follow-up duration did not differ significantly between groups (*p* = 0.326). Overall, BMI status was not associated with differences in perioperative parameters or postoperative bowel function over time (Table 2).

### 3.6. BMI-Based Longitudinal Changes in ODS, LARS and WIN Scores

ODS scores changed significantly over time (F-GG: F(1.82, 92.90) = 292.82, *p* < 0.001; partial η^2^ = 0.852). The time × BMI interaction was not significant (F-GG: F(1.82, 92.90) = 0.56, *p* = 0.556; partial η^2^ = 0.011), and no between-subjects main effect of BMI was observed (F(1, 51) = 1.17, *p* = 0.284; partial η^2^ = 0.022), indicating parallel postoperative recovery trajectories across BMI categories (Figure S7). LARS scores also decreased significantly over time (F-GG: F(1.28, 65.06) = 65.79, *p* < 0.001; partial η^2^ = 0.895). The time × BMI interaction was not significant (F-GG: F(1.28, 65.06) = 1.73, *p* = 0.194; partial η^2^ = 0.006), and no main effect of BMI was detected (F(1, 51) = 0.091, *p* = 0.765; partial η^2^ = 0.036), demonstrating similar postoperative recovery patterns in both BMI groups (Figure S8). WIS scores changed significantly over time (F-GG: F(1.95, 99.41) = 57.29, *p* < 0.001; partial η^2^ = 0.529), with an early postoperative increase followed by improvement toward POD365. The time × BMI interaction was not significant (F-GG: F(1.95, 99.41) = 1.83, *p* = 0.166; partial η^2^ = 0.035), and the between-subjects analysis showed no significant main effect of BMI (F(1, 51) = 0.511, *p* = 0.478; partial η^2^ = 0.010), indicating comparable longitudinal WIS trajectories across BMI categories (Figure S9).

### 3.7. Age-Based Comparative Analysis of Clinical and Functional Outcomes

Sex distribution did not differ between patients aged <50 years and ≥50 years (*p* = 0.333). ASA physical status differed between age groups, with a higher proportion of ASA I patients in the younger group (91.4%) and greater ASA II prevalence in patients aged ≥50 years (*p* < 0.001). Mean BMI (*p* = 0.458) and length of hospital stay (*p* = 0.707) were comparable between age groups. Preoperatively, younger patients had higher ODS scores (*p* = 0.005), whereas older patients had higher preoperative WIS scores (*p* < 0.001). Postoperatively, mean ODS scores at POD90 and POD180 were similar between groups, with a borderline difference at POD365 (*p* = 0.056). LARS scores were higher in older patients at POD180 (*p* = 0.016) and POD365 (*p* < 0.001). Postoperative WIS scores were lower in patients aged <50 years at POD90 (*p* < 0.001), POD180 (*p* < 0.001), and POD365 (*p* < 0.001). Follow-up duration was similar between age groups (*p* = 0.291) (Table 3).

### 3.8. Age-Based Longitudinal Changes in ODS, LARS and WIN Scores

ODS scores exhibited a pronounced and consistent change over time (F-GG adjusted; partial η^2^ = 0.847), reflecting a strong temporal trajectory characterized by significant linear, quadratic, and cubic components (all *p* < 0.001). This pattern differed modestly by age category, as evidenced by a significant time × age interaction limited to the quadratic component (F(1, 51) = 9.53, *p* = 0.003; partial η^2^ = 0.055), while linear and cubic interactions were not significant. Across the entire follow-up period, age category exerted a significant between-subject effect on overall ODS levels (F(1, 51) = 5.51, *p* = 0.023; partial η^2^ = 0.098), with older patients consistently demonstrating higher scores (Figure S10).

LARS scores followed a similar temporal evolution, showing a robust time effect (F-GG: F(1.27, 64.58) = 74.18, *p* < 0.001; partial η^2^ = 0.593). In contrast to ODS, the shape of this trajectory did not differ by age, as no significant time × age interaction was detected (F-GG: F(1.27, 64.58) = 0.39, *p* = 0.585; partial η^2^ = 0.008). Nevertheless, age category was associated with a persistently higher overall LARS burden (F(1, 51) = 7.12, *p* = 0.010; partial η^2^ = 0.123), indicating that older patients experienced worse symptoms throughout follow-up rather than at isolated time points (Figure S11).

WIS scores demonstrated marked temporal changes (F-GG: F(2.02, 102.76) = 83.00, *p* < 0.001; partial η^2^ = 0.619), accompanied by a significant time × age interaction (F-GG: F(2.02, 102.76) = 6.37, *p* = 0.002; partial η^2^ = 0.111). This interaction indicates that age not only influenced overall WIS levels but also modified the pattern of postoperative recovery over time. Consistently, age category showed a strong between-subject effect (F(1, 51) = 51.53, *p* < 0.001; partial η^2^ = 0.503), with substantially higher WIS scores observed in older patients across follow-up (Figure S12).

When adjusting for ASA class, the apparent association between age and postoperative LARS outcomes was attenuated and no longer independent (F = 0.338, *p* = 0.564, η^2^p = 0.007), whereas ASA class itself remained significantly associated with LARS severity (F = 4.815, *p* = 0.033, η^2^p = 0.089). No significant time × age (*p* = 0.702, η^2^p = 0.004) or time × ASA (*p* = 0.231, η^2^p = 0.030) interactions were observed, suggesting that ASA primarily influenced overall symptom burden rather than temporal patterns.

For ODS, a strong time effect persisted after adjustment (multivariate test: *p* < 0.001, η^2^p = 0.880), with neither age (F = 2.067, *p* = 0.157, η^2^p = 0.040) nor ASA class (F = 0.262, *p* = 0.611, η^2^p = 0.005) demonstrating independent between-subject effects, and no significant interactions. In contrast, WIS remained influenced by patient characteristics even after adjustment: although temporal change remained significant (F-GG-corrected: *p* < 0.001, η^2^p = 0.520) without significant time × age or time × ASA interactions, both age category (F = 9.137, *p* = 0.004, η^2^p = 0.157) and ASA class (F = 17.812, *p* < 0.001, η^2^p = 0.267) were independently associated with overall WIS scores, underscoring their sustained impact on continence-related outcomes.

### 3.9. Surgical Complications

Conversion to open surgery was required in one patient (1.88%) due to obesity and large uterine fibroids. One patient sustained a longitudinal rectal wall rupture during transanal specimen extraction because of an undivided mesosigmoid, requiring very low anterior resection. Four patients required early re-operation: two for pelvic abscesses (open drainage on POD 30; laparoscopic drainage on POD 8), one for laparoscopic repair of small-bowel herniation at a port site (POD 6), and one patient with mental retardation re-admitted on POD 18 with anastomotic leakage who died from pelvic sepsis. One patient developed transient premature ejaculation that resolved within eight months. Overall postoperative complication and mortality rates were 9.43% (*n* = 5) and 1.85% (*n* = 1), respectively.

### 3.10. Surgical Outcomes

Among 52 patients, 47 achieved complete anatomical improvement, while five developed postoperative prolapse-related recurrence: two with full-thickness prolapse (≥5 cm) and three with mucosal prolapse (≤2 cm). Median time to recurrence was 8 months (min–max: 3–13). The Delorme procedure was performed in two patients with full-thickness recurrence and three with mucosal prolapse (≤2 cm). The median time to both complete and mucosal recurrence was 8 months (min–max = 3–13).

### 3.11. Temporal Effects, Effect Sizes and Post Hoc Power Analysis

Repeated-measures general linear modeling demonstrated a significant main effect of time for all functional outcomes (ODS, LARS, and WIS). After F-GG correction, temporal effects remained significant for ODS (F = 322.8, *p* < 0.001; partial η^2^ = 0.861), LARS (F = 80.0, *p* < 0.001; partial η^2^ = 0.606), and WIS (F = 70.1, *p* < 0.001; partial η^2^ = 0.574). Based on observed effect sizes, sample size (n = 53), repeated measurements, and sphericity corrections (εODS = 0.606, εLARS = 0.633, εWIS = 0.641), post hoc analysis showed near-complete statistical power (1 − β ≈ 1.0) at α = 0.05.

## 4. Discussion

Complete RP is neither a uniform anatomical entity nor a homogeneous functional disorder of the pelvic floor; rather, it represents a complex condition that may be accompanied by prolapse of other pelvic segments, solitary rectal ulcer, fecal incontinence, and ODS. The resulting symptom burden often imposes substantial psychological stress and markedly reduces quality of life [50]. Accordingly, a personalized surgical strategy that optimizes both anatomical correction and functional recovery is preferable to a “one-size-fits-all” approach [1,6,19,22,23,27].

The overarching goal of surgical management is to restore rectal anatomy and pelvic floor physiology while improving continence- and constipation-related symptoms with acceptable mortality and recurrence rates. Regardless of approach, operative technique should be tailored to the individual patient. Although perineal procedures for RP are generally considered to better preserve pelvic autonomic function than some abdominal operations [51], extensive distal recto-anal dissection may still impair rectal capacity, anal canal sensitivity, and autonomic innervation of the posterior vaginal wall or prostate.

Abdominal sigmoid resection rectopexy for RP was first described by Frykman et al. in 1969 [52], with initially high anastomotic leakage rates (3.6%) [53]. LRR procedures were introduced after 1999 [23,24,26]. LRR combined with TSE has been reported in four studies. Can et al. [31] described the first LRR-TSE case in 2014, followed by a 25-patient series by Ates et al. [54] reporting satisfactory preliminary outcomes for constipation and incontinence but longer operative time and the need for at least one year of follow-up. Rajkumar et al. [55] subsequently reported a 16-patient series. Chen et al. [32] analyzed 45 patients (17 LRR-TSE, 28 conventional LRR), reporting shorter hospital stay but longer operative time with LRR-TSE. Apart from our own, only two patient series evaluating LRR-TSE in RP have been published

Division of the lateral ligaments that contain the neurovascular bundle may trigger new-onset constipation during the rectal mobilization required for rectal prolapse repair [56,57]. Several studies of resection rectopexy have sought to prevent this side-effect and improve functional outcomes [25,27,58]. The colonic transit time was improved in two studies in which patients underwent resection rectopexy [59]. In a study on 40 patients, Otto et al. [50] evaluated the functional results (using defecography) after a variety of rectal prolapse resections and found that constipation and incontinence scores improved. Thus, we preserved the lateral ligaments with neurovascular bundles in all patients and measured median ODS scores preoperatively and at three times postoperatively (the scores were 13, 2, 4, and 4, respectively, indicating gradual improvement; *p* < 0.001). No patient developed new or more severe ODS symptoms after surgery.

In the early postoperative period, some patients who underwent LRR-TSE reported high incontinence scores based on an increase in the frequency of unexplained bowel movements and, hence, the need to go to the toilet. These symptoms were considered to reflect transient LARS, as similarly described in the low anterior resection and LARS literature, and we thus derived LARS scores. This temporal pattern is consistent with previous reports indicating that most spontaneous improvement in LARS occurs within the first 6 months after surgery, with limited additional improvement thereafter [60]. We suggest that a minimum of 12 months of follow-up is required to obtain reliable, functional results, as LARS and new-onset incontinence may develop postoperatively [61]. We consider that the LRR operation stabilized the rectum and that excision of the excess sigmoid prevented rectal herniation and enabled the stool to reach the anal canal quickly. In the early postoperative period—thus, before the anal sphincter adapted to the anatomical correction—the increased frequency of bowel movements and the urgent need to go to the toilet caused patients to report high levels of incontinence and LARS-like symptoms. Ten of the patients (18.9%) exhibited no LARS symptoms; the three consecutive postoperative mean LARS scores were 18.0, 8.8, and 3.5, respectively (*p* < 0.001). The mean incontinence score improved after surgery from a preoperative score of 4.0, with postoperative scores of 8.1, 3.2, and 2.4, respectively (*p* < 0.001). LARS caused by the LRR procedure caused borderline deterioration (slight impairment) of anal continence in the early postoperative period; this overlapped with incontinence symptoms. Therefore, again, we believe that at least one year of follow-up data is required to evaluate the functional results accurately. The literature contains no data on the overlap between LARS and incontinence soon after rectal prolapse surgery [42,60,62].

When considering any resection rectopexy procedure, a significant concern is a risk posed by possible anastomotic complications. For LRR, a morbidity rate of 0–32% and a leakage rate of 0–2.5% have been reported in previous studies [26,27,63,64,65,66]. In the current study, three patients (9.43%) developed significant complications (two abscesses and one anastomotic leak). One patient (1.85%) died. Four studies, including 343 LRR patients, reported six deaths in total (from a cardiac condition in one, aspiration pneumonia in two, leakage-sepsis in one, and unknown causes in two); the mortality rate ranged from 1% to 6% [24,28,54,65].

The present study encountered three mucosal recurrences during 32 months of follow-up and two full-thickness rectal prolapse recurrences (3.77%). We did not score all mucosal prolapses as recurrences because a definitive definition of a recurrent prolapse is lacking, and it is not clear that a mucosal prolapse developing after surgery should be regarded as a recurrence. In the literature, the average operation time of LRR for treating rectal prolapse was 110–258 min [24,26]. In the present study, the median operation time was 182 (140–245) min, although all procedures were performed by an experienced colorectal surgeon; this time is longer than that reported in the literature. During LRR-TSE, unlike other laparoscopic procedures, division of the mesorectum and anal extraction increases the operation time.

However, laparoscopic colorectal surgery remains imperfect; an abdominal incision with a minimum length of 5–7 cm is required to remove the specimen. Complications remain, including infection of the abdominal incision, postoperative somatic pain, and incisional hernia [67]. Winslow and colleagues found that, after laparoscopic colon surgery, wound infections and incisional hernias at the extraction site developed in 10.8% and 21.6% of patients, respectively [68]. The potential benefits of applying NOSE during colorectal surgery include reductions in postoperative pain and wound complications, a lower requirement for postoperative analgesia, faster recovery of bowel function, a shorter hospital stay, and better cosmetic and psychological outcomes [69]. Although NOSE significantly reduces surgical trauma, the technique features various pitfalls. Bacteriological concerns have been raised [70]. Nevertheless, although bacterial growth has been observed in peritoneal culture samples, no infectious complications have been reported [69,70]. In the current study, despite prophylactic antibiotic administration, mechanical bowel preparation, intraoperative peritoneal irrigation, and rectal lavage, two patients (3.7%) developed retrorectal abscesses that did not allow percutaneous drainage and thus required re-operation. Bacterial contamination might have occurred during the LRR-TSE procedure.

One of the most debated issues in the surgical management of rectal prolapse is whether an abdominal or a perineal approach should be preferred. Surgical management of complete rectal prolapse is therefore centered on these two principal strategies, and contemporary evidence indicates that the choice between them should be individualized rather than hierarchical. Evidence consistently shows that perineal procedures, including Altemeier’s and Delorme’s operations, are associated with shorter operative time and reduced perioperative physiological stress, as well as shorter hospital stay, making them attractive options for elderly or frail patients. However, these advantages are counterbalanced by higher long-term recurrence rates, which are commonly reported in the range of approximately 20.1–27.9%, and by less predictable improvement in functional outcomes. In contrast, abdominal procedures—such as suture rectopexy, mesh rectopexy, and resection rectopexy—are generally associated with longer operative duration and hospital stay, reflecting greater technical complexity and operative burden. Nevertheless, they provide more durable anatomical correction, with lower recurrence rates typically reported between approximately 7.7–15.6%, particularly with longer follow-up. Functional outcomes also differ between approaches: abdominal surgery is overall associated with a lower risk of postoperative fecal incontinence, whereas constipation may persist or worsen in some patients, especially when sigmoid resection is not performed [11,71,72,73,74]. With the adoption of laparoscopic techniques in abdominal rectal prolapse surgery, wound-related complications, postoperative pain, recurrence and functional results have improved compared with conventional open and perineal approaches [75,76]. Taken together, these data highlight a clear trade-off between operative burden and long-term durability, supporting a tailored, center- and surgeon-specific strategy in which recurrence risk, operative time, continence, and length of stay are balanced against patient characteristics and institutional expertise when selecting the optimal surgical approach.

### Limitations

This study has several limitations that should be acknowledged. First, the relatively small sample size and single-center design may limit the generalizability of the findings. However, the robustness of the observed temporal changes is supported by the large within-subject effect sizes and post hoc power analyses, indicating sufficient statistical power to detect clinically meaningful differences despite the limited sample size. Second, the absence of a control group undergoing conventional laparoscopic resection rectopexy without transanal specimen extraction precludes a direct assessment of the incremental contribution of the NOSE component; therefore, the present study cannot determine whether observed benefits in postoperative recovery or functional outcomes are uniquely attributable to LRR-TSE. Third, the lack of more detailed preoperative functional assessments and advanced pelvic floor imaging may have limited baseline characterization and reduced the ability to identify predictors of postoperative functional outcomes. Fourth, although data were collected prospectively, analyses were performed retrospectively after completion of the observation period, which is inherently associated with limitations such as unmeasured confounding and potential selection bias. Future studies involving larger, multicenter cohorts with appropriate comparator arms and standardized preoperative functional and imaging assessments are warranted to further clarify the safety profile, functional implications, and potential advantages of LRR-TSE over standard laparoscopic techniques.

## 5. Conclusions

This study demonstrates that LRR-TSE is a feasible minimally invasive option for the treatment of complete RP, with acceptable functional and recurrence outcomes in carefully selected patients. The technique provides substantial improvements in constipation, continence, and LARS-related bowel dysfunction, as evidenced by significant longitudinal reductions in ODS, WIS, and LARS scores across the entire cohort. These functional benefits became more reliable after one year, when the confounding influence of early postoperative LARS diminished and long-term bowel function stabilized. Given the observational design and the limited availability of comparative evidence, prospective randomized studies comparing LRR-TSE with alternative surgical techniques using standardized outcome measures are warranted to better define its role in clinical practice.

## Figures and Tables

**Table 1:** Sex-based comparison of demographic, clinical, and functional outcomes after laparoscopic RP surgery.

Parameters	Statistical Features	Female	Male	Total	*p*
Age	Mean (SD)	48 (12)	39 (16)	45 (14)	0.024
	95%CI	[44,45,46,47,48,49,50,51,52]	[32,33,34,35,36,37,38,39,40,41,42,43,44,45,46]	[41,42,43,44,45,46,47,48]	
ASA Score	ASA I	20 (62.5)	16 (76.2)	36 (67.9)	0.374
	ASA II	12 (37.5)	5 (23.8)	17 (32.1)	
BMI	Mean (SD)	27.4 (3.6)	26.8 (3.5)	27.1 (3.5)	0.544
	95%CI	[26.1–28.7]	[25.2–28.3]	[26.1–28.1]	
Hospital Stay	Mean (SD)	5 (2)	6 (5)	5 (4)	0.529
	95%CI	[4,5,6]	[3,4,5,6,7,8]	[4,5,6]	
ODS (Preop)	Mean (SD)	12.2 (3.2)	13.6 (2.9)	12.8 (3.2)	0.125
	95%CI	[11.1–13.4]	[12.3–15.0]	[11.9–13.7]	
ODS (POD90)	Mean (SD)	2.7 (2.5)	1.9 (1.2)	2.4 (2.1)	0.148
	95%CI	[1.8–3.6]	[1.4–2.4]	[1.8–2.9]	
ODS (POD180)	Mean (SD)	4.3 (2.3)	4.1 (2.1)	4.2 (2.2)	0.732
	95%CI	[3.5–5.2]	[3.2–5.0]	[3.6–4.8]	
ODS (POD365)	Mean (SD)	5.2 (3.1)	5.2 (2.6)	5.2 (2.9)	0.981
	95%CI	[4.1–6.3]	[4.0–6.4]	[4.4–6.0]	
LARS (POD90)	Mean (SD)	18.6 (13.2)	17.1 (12.1)	18.0 (12.7)	0.694
	95%CI	[13.8–23.3]	[11.6–22.7]	[14.5–21.5]	
LARS (POD180)	Mean (SD)	9.0 (6.9)	8.6 (6.6)	8.8 (6.8)	0.836
	95%CI	[6.5–11.5]	[5.6–11.6]	[6.9–10.7]	
LARS (POD365)	Mean (SD)	4.0 (4.8)	2.9 (3.3)	3.5 (4.2)	0.341
	95%CI	[2.3–5.7]	[1.4–4.3]	[2.4–4.7]	
WIS (Preop)	Mean (SD)	4.0 (4.0)	3.9 (3.7)	4.0 (3.9)	0.874
	95%CI	[2.6–5.5]	[2.2–5.5]	[2.9–5.0]	
WIS (POD90)	Mean (SD)	9.2 (5.2)	6.2 (4.6)	8.1 (5.2)	0.037
	95%CI	[7.4–11.1]	[4.2–8.3]	[6.6–9.5]	
WIS (POD180)	Mean (SD)	3.5 (4.3)	2.6 (2.8)	3.2 (3.7)	0.407
	Min-Max	[2.0–5.0]	[1.3–3.9]	[2.1–4.2]	
WIS (POD365)	Mean (SD)	2.6 (3.3)	2.1 (2.5)	2.4 (3.0)	0.573
	95%CI	[1.4–3.8]	[1.0–3.3]	[1.6–3.3]	
Follow up (mo)	Mean (SD)	77.4 (19.9)	83.0 (18.7)	79.6 (19.5)	0.312
	95%CI	[70.2–84.6]	[74.4–91.5]	[74.2–85.0]	

**Table 2:** BMI-based comparison of demographic, clinical, and functional outcomes after laparoscopic RP surgery.

Parameters	Statistical Features	BMI < 25	BMI ≥ 25	Total	*p*
Age	Mean (SD)	45 (14)	44 (14)	45 (14)	0.739
	95%CI	[39,40,41,42,43,44,45,46,47,48,49,50,51,52]	[39,40,41,42,43,44,45,46,47,48,49]	[41,42,43,44,45,46,47,48]	
Gender	Male	9 (50)	12 (34.3)	21 (39.6)	0.417
	Female	9 (50)	23 (65.7)	32 (60.4)	
ASA Score	ASA I	12 (66.7)	24 (68.6)	36 (67.9)	1.000
	ASA II	6 (33.3)	11 (31.4)	17 (32.1)	
Hospital Stay	Mean (SD)	6 (6)	5 (2)		0.148
	95%CI	[3,4,5,6,7,8,9]	[4,5]		
ODS (Preop)	Mean (SD)	13.6 (3.6)	12.4 (2.9)	12.8 (3.2)	0.212
	95%CI	[11.8–15.3]	[11.4–13.4	[11.9–13.7]	
ODS (POD90)	Mean (SD)	2.8 (2.8)	2.1 (1.6)	2.4 (2.1)	0.301
	95%CI	[1.4–4.2]	[1.6–2.7]	[1.8–2.9]	
ODS (POD180)	Mean (SD)	4.7 (2.5)	4.0 (2.0)	4.2 (2.2)	0.306
	95%CI	[3.4–5.9]	[3.3–4.7]	[3.6–4.8]	
ODS (POD365)	Mean (SD)	5.3 (3.0)	5.2 (2.9)	5.2 (2.9)	0.850
	95%CI	[3.9–6.8]	[4.2–6.2]	[4.4–6.0]	
LARS (POD90)	Mean (SD)	16.0 (12.0)	19.0 (13.1)	18.0 (12.7)	0.415
	95%CI	[10.1–21.9]	[14.5–23.5]	[14.5–21.5]	
LARS (POD180)	Mean (SD)	8.6 (6.9)	8.9 (6.8)	8.8 (6.8)	0.879
	95%CI	[5.2–12.0]	[6.6–11.3]	[6.9–10.7]	
LARS (POD365)	Mean (SD)	4.5 (5.2)	3.1 (3.6)	3.5 (4.2)	0.243
	95%CI	[1.9–7.1]	[1.8–4.3]	[2.4–4.7]	
WIS (Preop)	Mean (SD)	4.6 (4.2)	3.7 (3.7)	4.0 (3.9)	0.429
	95%CI	[2.5–6.7]	[2.4–4.9]	[2.9–5.0]	
WIS (POD90)	Mean (SD)	7.7 (4.9)	8.2 (5.4)	8.1 (5.2)	0.739
	95%CI	[5.3–10.2]	[6.4–10.1]	[6.6–9.5]	
WIS (POD180)	Mean (SD)	4.1 (4.6)	2.7 (3.2)	3.2 (3.7)	0.210
	95%CI	[1.8–6.3]	[1.6–3.8]	[2.1–4.2]	
WIS (POD365)	Mean (SD)	3.2 (3.7)	2.0 (2.5)	2.4 (3.0)	0.173
	95%CI	[1.4–5.1]	[1.2–2.9]	[1.6–3.3]	
Follow up (mo)	Mean (SD)	75.9 (17.2)	81.5 (20.5)	79.6 (19.5)	0.326
	95%CI	[67.3–84.4]	[74.4–88.5]	[74.2–85.0]	

**Table 3:** Age-based comparison of demographic, clinical, and functional outcomes after laparoscopic RP surgery.

Parameters	Statistical Features	Age < 50 yr	Age ≥ 50 yr	Total	*p*
Gender	Male	16 (45.7)	5 (27.8)	21 (39.6)	0.333
	Female	19 (54.3)	13 (72.2)	32 (60.4)	
ASA Score	ASA I	32 (91.4)	4 (22.2)	36 (67.9)	<0.001
	ASA II	3 (8.6)	14 (77.8)	17 (32.1)	
BMI	Mean (SD)	27.4 (3.8)	26.6 (2.9)	27.1 (3.5)	0.458
	95%CI	[26.1–28.7]	[25.2–28.1]	[26.1–28.1]	
Hospital Stay	Mean (SD)	5 (4)	5 (2)	5 (4)	0.707
	95%CI	[4,5,6,7]	[4,5,6]	[4,5,6]	
ODS (Preop)	Mean (SD)	13.7 (2.9)	11.1 (3.0)	12.8 (3.2)	0.005
	95%CI	[12.7–14.7]	[9.6–12.6]	[11.9–13.7]	
ODS (POD90)	Mean (SD)	2.5 (2.2)	2.0 (2.0)	2.4 (2.1)	0.377
	95%CI	[1.8–3.3]	[1.0–3.0]	[1.8–2.9]	
ODS (POD180)	Mean (SD)	4.5 (2.2)	3.7 (2.3)	4.2 (2.2)	0.240
	95%CI	[3.7–5.2]	[2.6–4.9]	[3.6–4.8]	
ODS (POD365)	Mean (SD)	5.8 (2.8)	4.2 (2.8)	5.2 (2.9)	0.056
	95%CI	[4.8–6.7]	[2.8–5.6]	[4.4–6.0]	
LARS (POD90)	Mean (SD)	15.8 (13.0)	22.3 (11.1)	18.0 (12.7)	0.078
	95%CI	[11.3–20.3]	[16.8–27.8]	[14.5–21.5]	
LARS (POD180)	Mean (SD)	7.2 (6.6)	11.9 (6.2)	8.8 (6.8)	0.016
	95%CI	[5.0–9.5]	[8.8–15.0]	[6.9–10.7]	
LARS (POD365)	Mean (SD)	2.0 (3.0)	6.6 (4.8)	3.5 (4.2)	<0.001
	95%CI	[1.0–3.0]	[4.2–8.9]	[2.4–4.7]	
WIS (Preop)	Mean (SD)	2.5 (2.8)	6.9 (4.0)	4.0 (3.9)	<0.001
	95%CI	[1.5–3.4]	[4.9–8.9]	[2.9–5.0]	
WIS (POD90)	Mean (SD)	5.5 (3.5)	13.0 (4.2)	8.1 (5.2)	<0.001
	95%CI	[4.3–6.7]	[10.9–15.1]	[6.6–9.5]	
WIS (POD180)	Mean (SD)	1.5 (1.8)	6.4 (4.4)	3.2 (3.7)	<0.001
	95%CI	[0.8–2.1]	[4.3–8.6]	[2.1–4.2]	
WIS (POD365)	Mean (SD)	1.0 (1.2)	5.2 (3.5)	2.4 (3.0)	<0.001
	95%CI	[0.6–1.5]	[3.4–6.9]	[1.6–3.3]	
Follow up (mo)	Mean (SD)	77.3 (16.4)	84.1 (24.2)	79.6 (19.5)	0.291
	95%CI	[71.6–82.9]	[72.1–96.2]	[74.2–85.0]	

## Data Availability

The datasets analyzed during the current study are available from the corresponding author upon reasonable request.

## Supplementary Materials

The following supporting information can be downloaded at https://www.mdpi.com/article/10.3390/jcm15020718/s1: Figure S1: Temporal changes in ODS scores in the entire study cohort from preoperative assessment to 12-month follow-up; Figure S2: Temporal changes in LARS scores in the entire study cohort across postoperative follow-up intervals; Figure S3: Temporal changes in WIS scores in the entire study cohort from baseline to long-term postoperative evaluation; Figure S4: Sex-based comparison of longitudinal changes in ODS scores; Figure S5: Sex-based comparison of postoperative LARS trajectories; Figure S6: Sex-based comparison of longitudinal WIS score changes; Figure S7: BMI-based comparison of longitudinal ODS score changes; Figure S8: BMI-based comparison of postoperative LARS score trajectories; Figure S9: BMI-based comparison of longitudinal WIS score changes; Figure S10: Age-related differences in postoperative ODS score trajectories; Figure S11: Age-related comparison of LARS scores across follow-up intervals; Figure S12: Age-related comparison of longitudinal WIS score changes.
