# Supplementary material for: Laparoscopic Resection Rectopexy with Transanal Specimen Extraction for Complete Rectal Prolapse: Retrospective Cohort Study of Functional Outcomes
Source: J Clin Med. 2026 Jan 15;15(2):718. doi: 10.3390/jcm15020718 (PMC12842517; doi:10.3390/jcm15020718)
Supplement: Supplementary file 1 [file jcm-15-00718-s001.zip › jcm-4064185-supplementary.pdf]

Laparoscopic Resection Rectopexy with Transanal Specimen Extraction for Complete Rectal Prolapse: Retrospective Cohort Study of Functional Outcomes

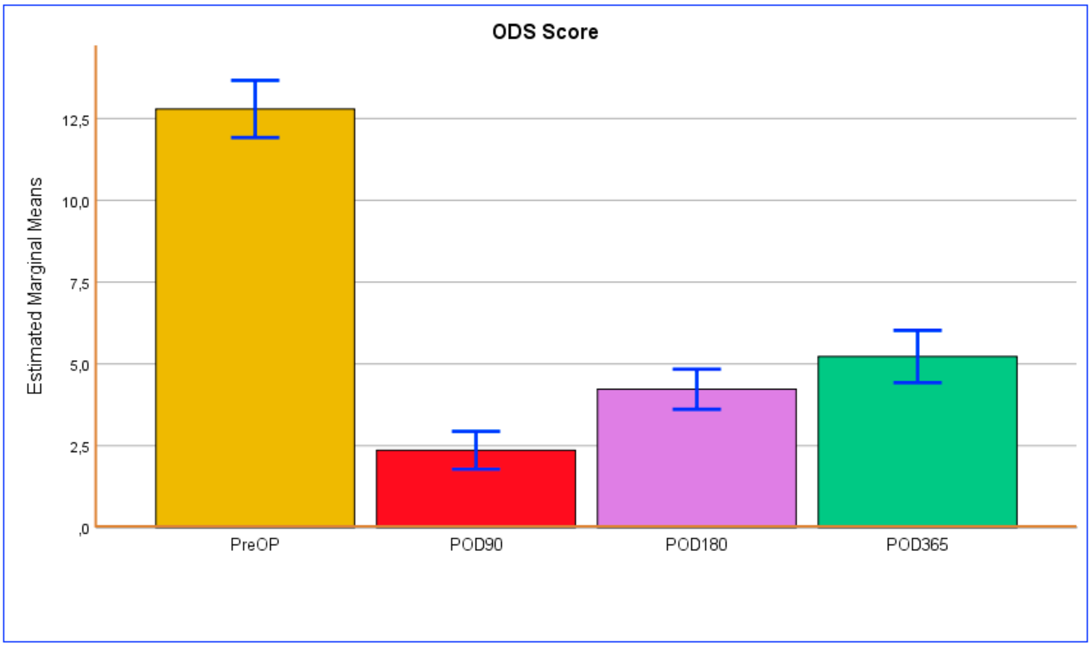

Figure S1. Temporal changes in ODS scores in the entire study cohort from preoperative assessment to 12-month follow-up.

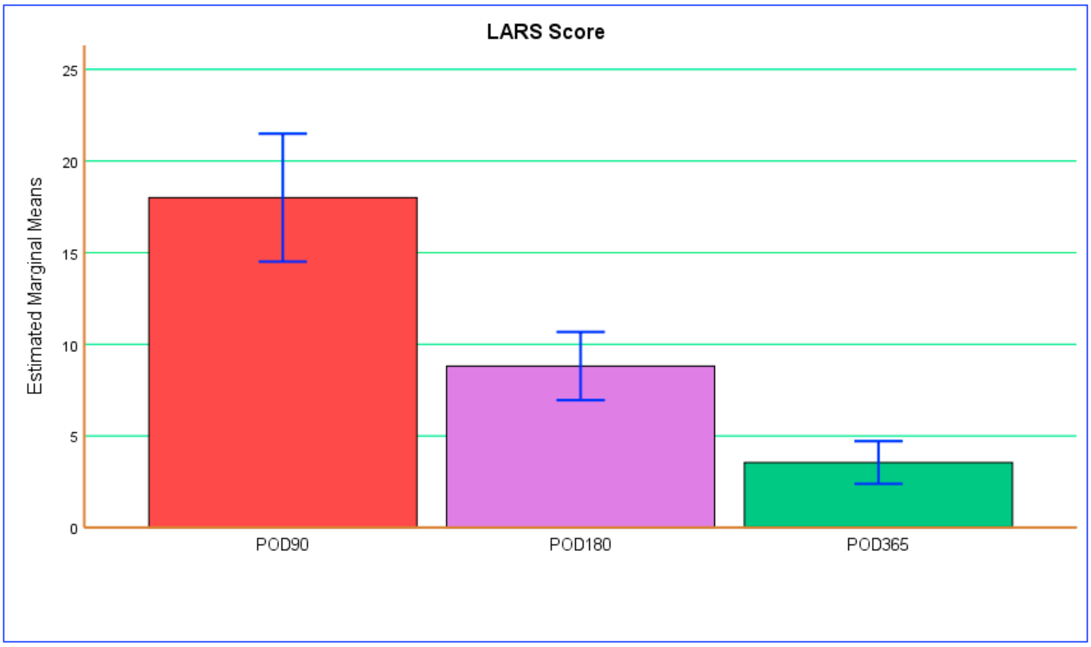

Figure S2. Temporal changes in LARS scores in the entire study cohort across postoperative follow-up intervals.

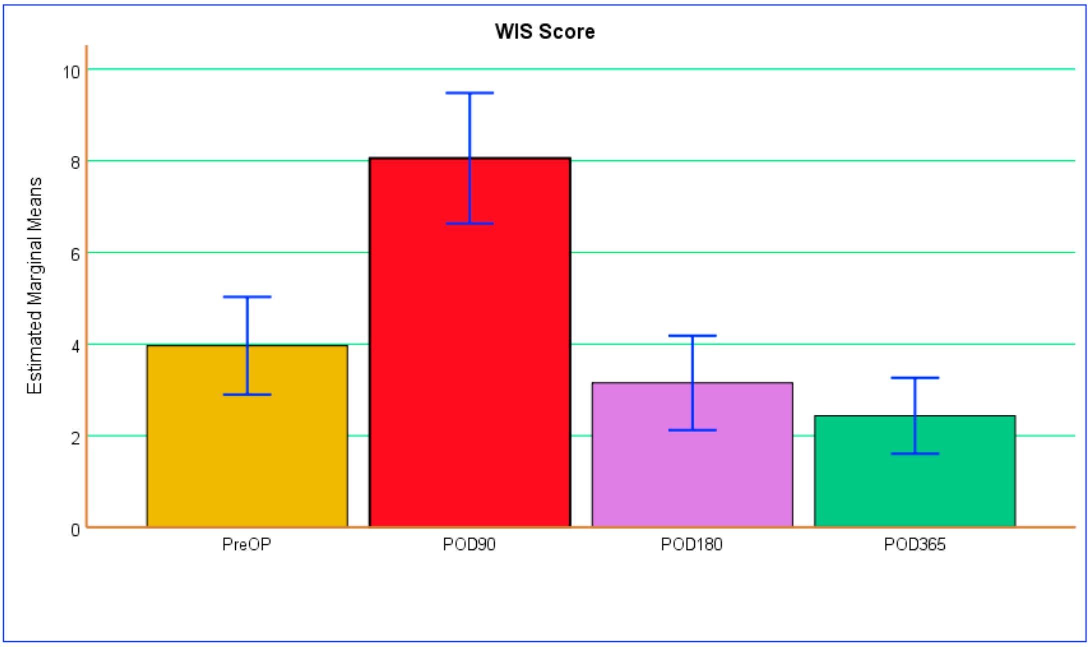

Figure S3. Temporal changes in WIS scores in the entire study cohort from baseline to long-term postoperative evaluation.

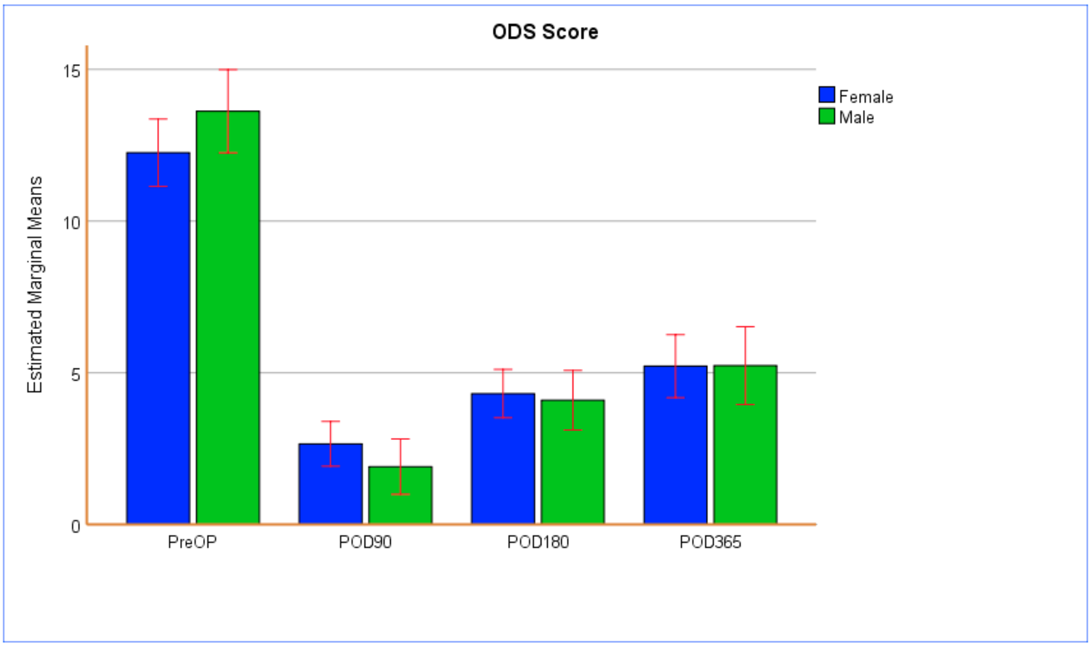

Figure S4. Sex-based comparison of longitudinal changes in ODS scores.

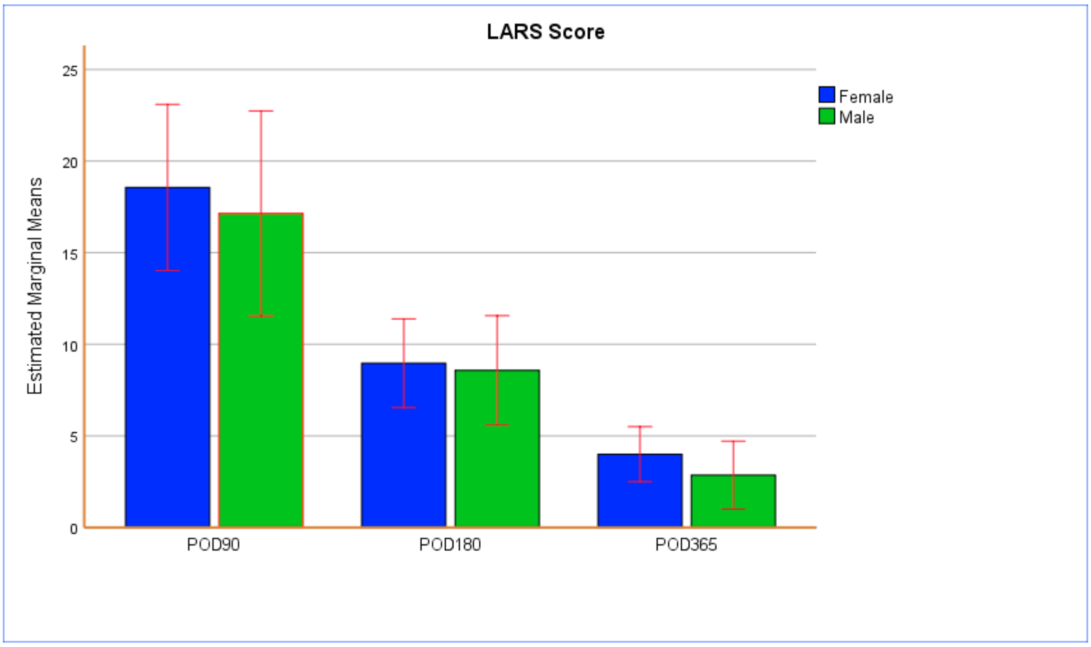

Figure S5. Sex-based comparison of postoperative LARS trajectories.

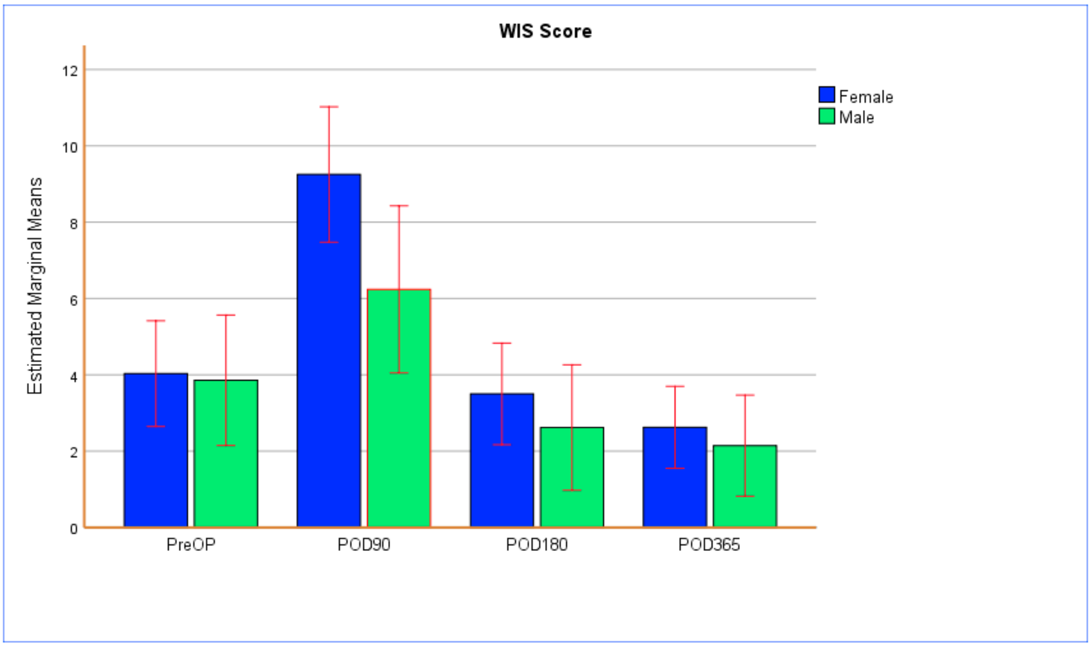

Figure S6. Sex-based comparison of longitudinal WIS score changes.

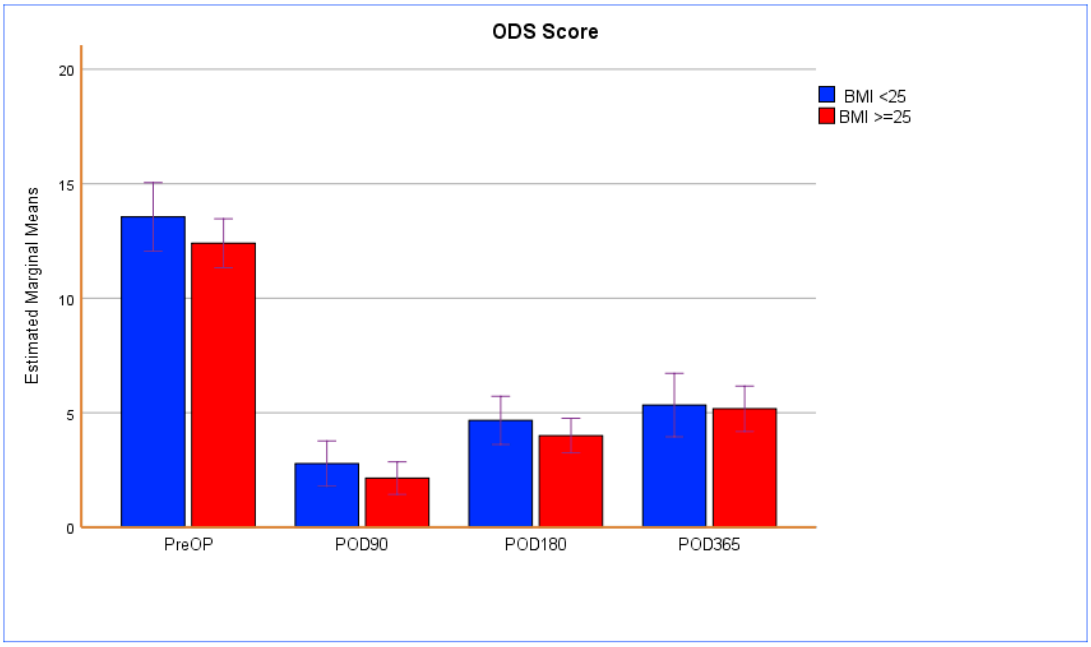

Figure S7. BMI-based comparison of longitudinal ODS score changes.

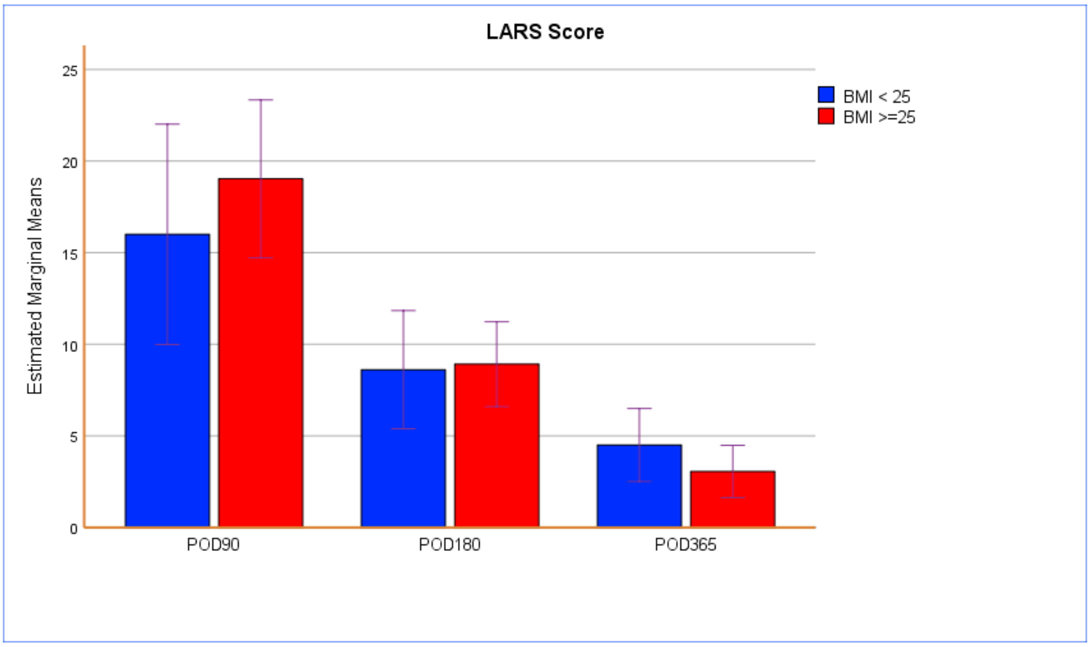

Figure S8. BMI-based comparison of postoperative LARS score trajectories.

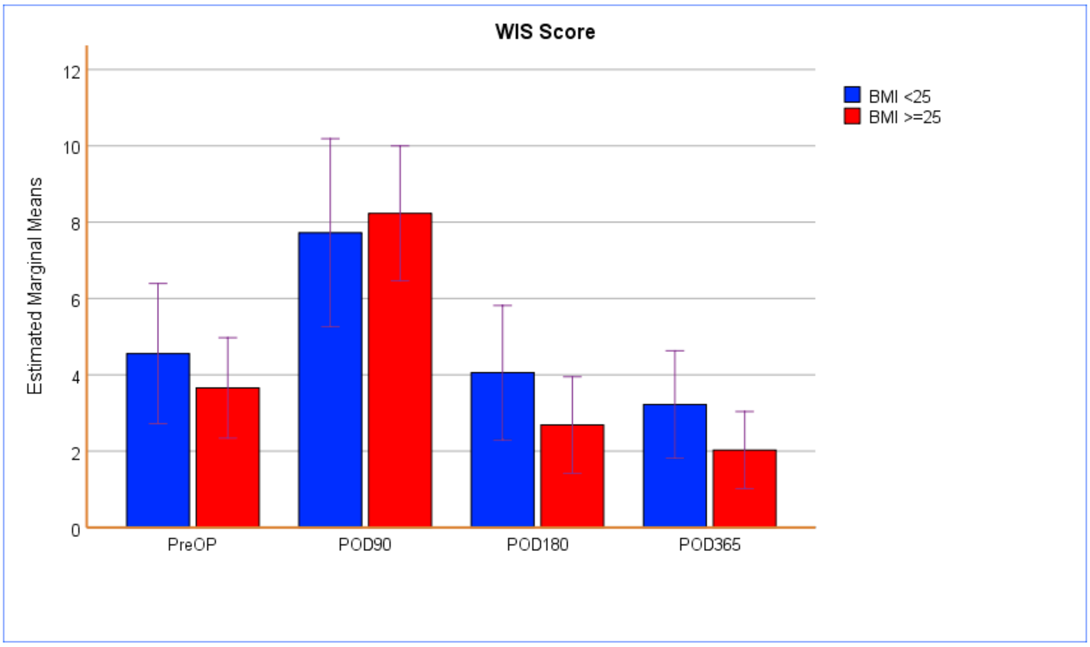

Figure S9. BMI-based comparison of longitudinal WIS score changes.

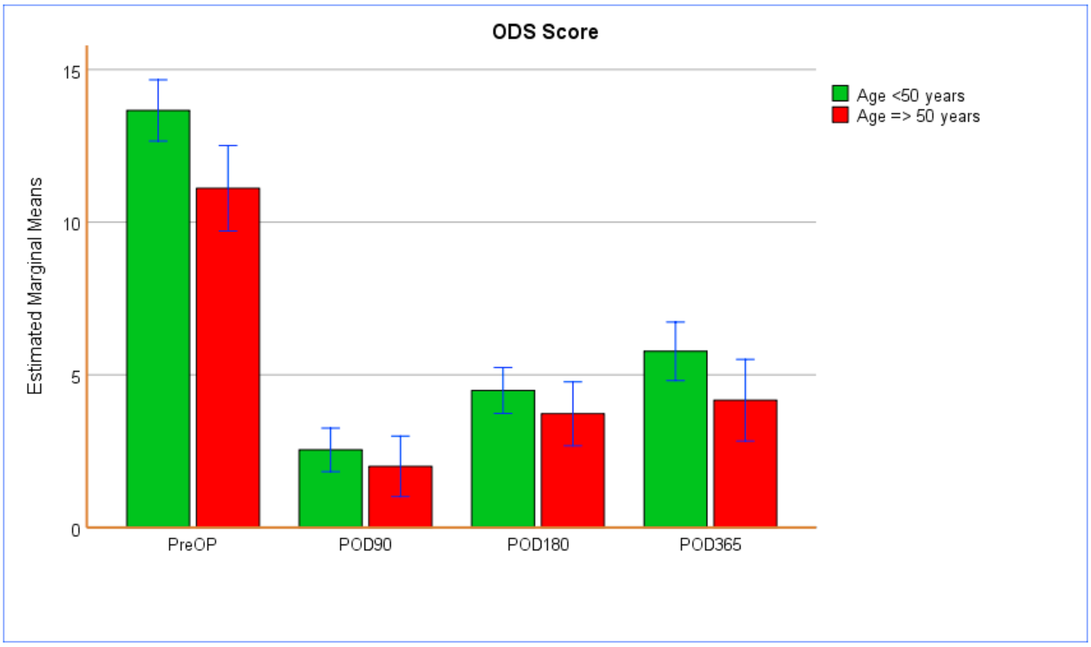

Figure S10. Age-related differences in postoperative ODS score trajectories

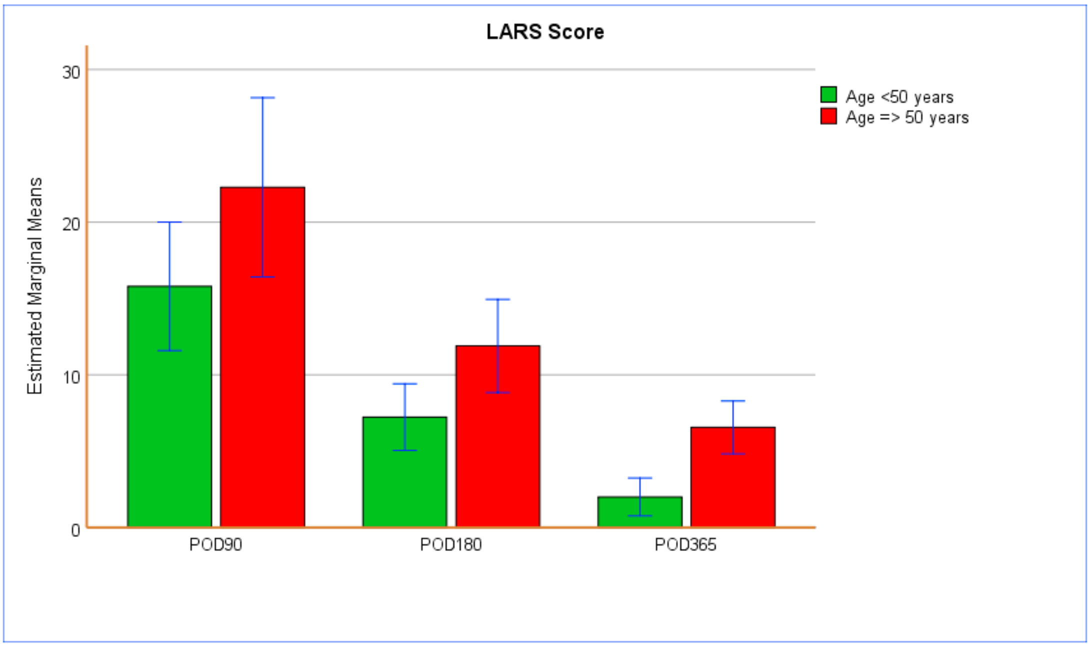

Figure S11. Age-related comparison of LARS scores across follow-up intervals.

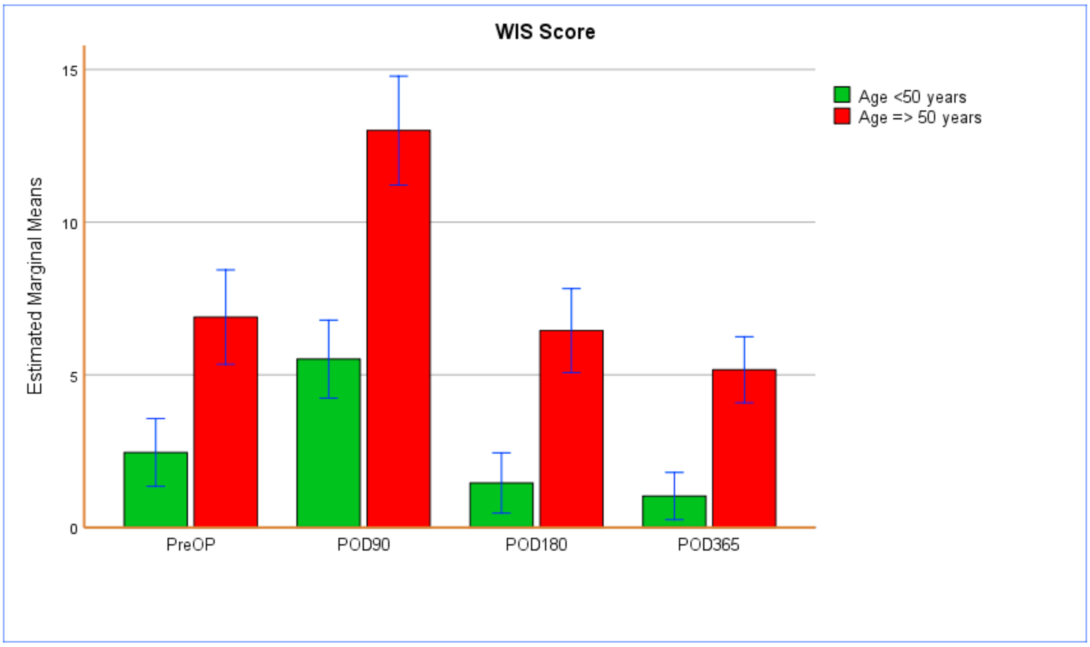

Figure S12. Age-related comparison of longitudinal WIS score changes.
